# Comparison of the Effect of Mouthwashes with and without Zinc and Fluocinolone on the Healing Process of Erosive Oral Lichen Planus

**DOI:** 10.5681/joddd.2010.007

**Published:** 2010-03-14

**Authors:** Masoumeh Mehdipour, Ali Taghavi Zenouz, Aila Bahramian, Javad Yazdani, Firoz Pouralibaba, Katayoun Sadr

**Affiliations:** ^1^ Assistant Professor, Department of Oral Medicine, Faculty of Dentistry, Tabriz University of Medical Sciences, Tabriz, Iran; ^2^ Post-graduate Student, Department of Oral Medicine, Faculty of Dentistry, Tabriz University of Medical Sciences, Tabriz, Iran; ^3^ Assistant Professor, Department of Maxillofacial Surgery, Faculty of Dentistry, Tabriz University of Medical Sciences, Tabriz, Iran; ^4^ Assistant Professor, Department of Prosthodontics, Faculty of Dentistry, Tabriz University of Medical Sciences, Tabriz, Iran

**Keywords:** Fluocinolone ointment, oral lichen planus, zinc mouthwash

## Abstract

**Background and aims:**

Lichen planus is a chronic inflammatory disorder with unspecified etiology, appearing as a result of stress, genetic predisposition and immunologic factors. Erosive type of the disease is more important because of its clinical symptoms of pain, irritation and malignancy risk. Despite various medications used, a definite cure for lichen planus is un-known. Regarding the effect of zinc on healing of ulcers, the aim of this study was to compare the effect of a mouthwash with and without zinc and fluocinolone on healing of erosive oral lichen planus.

**Materials and methods:**

Twenty randomly-selected patients with erosive oral lichen planus were divided into two groups of 10. One group received zinc mouthwash with fluocinolone ointment and the other group received placebo with ointment. The largest dimension of the ulcers was measured by digital calipers and the intensity of pain was determined by visual ana-logue scale. Data was analyzed with Mann-Whitney U test.

**Results:**

Pain, irritation and lesion surface area decreased in both groups. Decrease in pain severity was identical in both groups (P = 0.11). However, decrease in surface area with zinc mouthwash plus fluocinolone was more than that with only fluocinolone (P = 0.037).

**Conclusion:**

0.2% zinc mouthwash plus fluocinolone and only fluocinolone were both effective in decreasing pain, irrita-tion, and surface area of OLP. However, decrease in surface area with zinc mouthwash plus fluocinolone was more than that with fluocinolone alone.

## Introduction

Lichen planus is a chronic inflammatory disease which involves skin and mucous membranes, with a prevalence of 0.5 -2.2% in the general population. Although the etiology is unknown, it is believed that stress, heredity and immunologic factors are effective in this regard. Oral lichen planus (OLP) lesions have been divided into reticular, atrophic, erosive and bullous forms. Although the erosive form is not as prevalent as the reticular form, it is more important for patients because of its clinical symptoms. Despite the use of various medications such as corticosteroids, retinoids, cyclosporine, Dapson and other drugs with various side effects, a definite cure for lichen planus is still unknown.^1^

In recent studies on healing processes of ulcers, the role of trace elements, such as zinc, has been examined. Topical use of zinc results in regeneration of epithelium and repair of the endothelium of vessels. The role of zinc compounds in strengthening local defense system, which reduces inflammation and bacterial growth, has been demonstrated in regeneration of foot injuries.^2^ Zinc serves as a cofactor in numerous transcription factors and enzyme systems, including zinc-dependent matrix metalloproteinases that augment auto-debridement and keratinocyte migration during wound repair.^3^

It has been reported that prescription of zinc compounds simultaneous with steroids, reduces chronic eczema, lichen planus and limited psoriasis symptoms.^4^ It has also been reported that topically applied zinc compounds can reduce pain, itching, duration of infection and recurrence of herpetic ulcers.^5^ Few studies have been carried out on the topical effect of zinc on erosive OLP. In this study the effect of zinc mouthwash was compared with ordinary treatment of lichen planus, which consists of prescription of topical steroids.

## Materials and methods

This random double-blind study was performed on patients with erosive and atrophic OLP referring to the Department of Oral Medicine, Faculty of Dentistry, Tabriz University of Medical Sciences. The diagnosis of OLP was based on clinical characteristics including presence of Wikham’s striae and lesions on both cheeks. Biopsy confirmed the symptomatic OLP. The exclusion criteria were the following: pregnancy; sensitivity to zinc mouthwash or fluocinolone ointment; systemic use of corticosteroids; uncontrolled diabetes; history of tuberculosis, peptic ulcers and high blood pressure; use of medications during the previous month for treatment of lichen planus and hypertension; tobacco use; and lesions in contact with corroding dental amalgam.

Patients participated voluntarily and completed informed consent forms. Twenty consecutive patients diagnosed with erosive and atrophic OLP were randomly divided into two groups of 10. At baseline, the patients were carefully examined. The largest dimension of the lesion was determined with digital calipers and pain severity was determined with visual analogue scale (VAS).^1^ Then treatment was initiated in the following manner: The patients underwent treatment without knowing to which group they belonged; however, they used mouthwash A or B (A: placebo mouthwash; B: 0.2% zinc mouthwash) 3 times a day (morning, noon and night) and fluocinolone twice a day (morning and night) for 2 months from the baseline.

The subjects used 10 mL of mouthwash for one minute 3 times a day and abstained from eating and drinking for one hour; then the mouth was rinsed with water, and fluocinolone ointment was applied on the oral lesions immediately so that the affected area was completely covered with the drug. Then the subjects abstained from eating and drinking for 15 minutes and then the oral cavity was rinsed with water. The patients were examined at weekly intervals during the first month and at two-weekly intervals during the second month. VAS was used for assessing pain severity caused by the lesion and digital calipers were used for evaluating the improvement rate of the lesion. Data were analyzed with Mann-Whitney U test using SPSS software.^6^

## Results

The average scores of pain severity were 3.77 and 5.22 in the groups receiving mouthwashes A and B, respectively, with no significant difference between the two groups (P = 0.283). According to the results, during the seven sessions that followed, there were no significant differences in the decreasing trend of pain severity and irritation between the two groups (P = 0.11); however, this trend was significant in each group separately (P = 0.00), which shows that the pain severity score decreased in the two groups (P = 0.00) (Figure 1).

**Figure:**
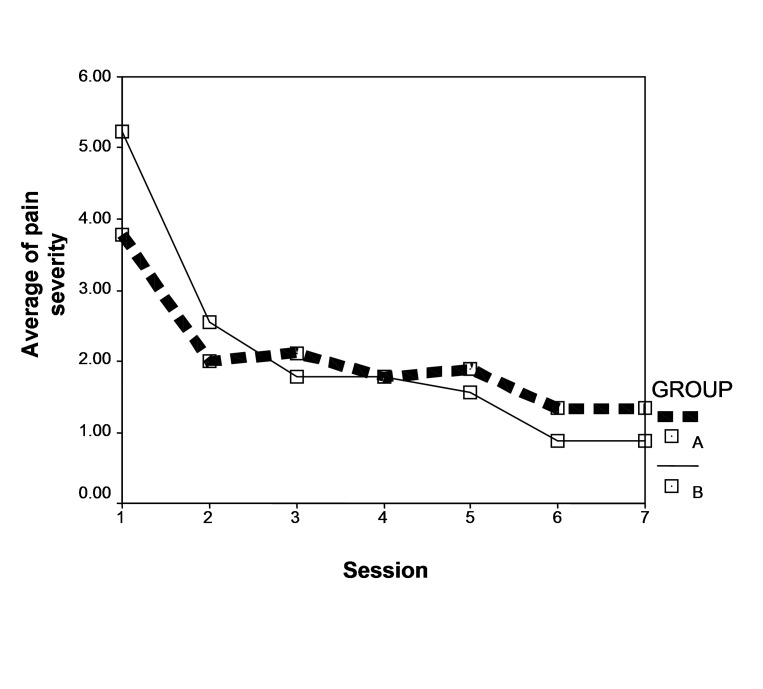
Figure 1. Comparison of decreasing trend in pain intensity between the placebo mouthwash group (A) and zinc mouthwash group (B).

At baseline, the largest diameter of erosive lesion was measured and recorded in both groups, with the average scores of 19.86 mm and 31.67 mm in the groups receiving mouthwashes B and A, respectively, demonstrating no significant differences (P = 0.691). Therefore, both groups had the same lesion size at the beginning of the study. According to the results, reduction trend of lesion size was not significant between the two groups (P = 0.132). This trend was not significant in each group separately, either (P = 0.11).

At baseline, the largest diameter of reticular lesion was measured and recorded in both groups, with the average scores of 21.25 and 22.27 in the groups receiving mouthwashes B and A, respectively, demonstrating no significant differences (P = 0.75). Therefore, each group had the same measurement of reticular lesion at the beginning of the study. The results showed no significant differences between the two groups in decreasing the trend of reticular lesions’ size (P = 0.333). This trend was not significant in each group, separately, either (P = 0.668).

At baseline, approximate surface areas of erosive, reticular and erythematous lesions were measured separately: the largest dimension of the lesion was multiplied by the smallest dimension of the lesion. Piboonniyom formula was used to this end.

At baseline, average scores were 6.77 and 5.22 in the groups receiving mouthwashes B and A, respectively, with no significant differences (P = 0.69). In other words, the two groups had the same score at baseline. According to the results, score improvement trend was significant between the two groups (P = 0.037); in addition, this trend was significant in each group (P = 0.012) (Figure 2).

**Figure:**
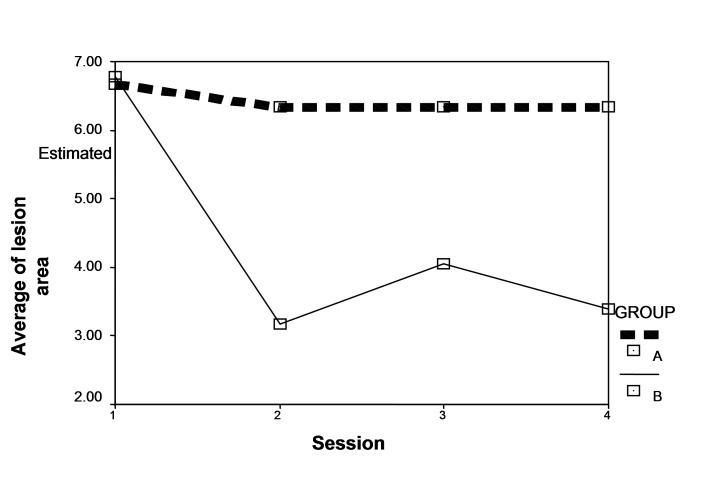
Figure 2. Comparison of decreasing trend in lesion surface area between the placebo mouthwash group (A) and zinc mouthwash group (B).

## Discussion

According to the results of the present study, either zinc mouthwash with fluocinolone ointment or fluocinolone ointment separately was effective in decreasing lesion surface area, pain, and irritation of erosive oral lichen planus. Decrease in pain and irritation in both groups was identical, which might be attributed to the effect of fluocinolone ointment used in both groups. However, decrease in surface area with zinc mouthwash plus ointment was more than that with ointment alone, which is attributable to the effect of zinc on healing of disrupted epithelium.

Buajeeb et al,^7^ comparing the efficacy of topical retinoic acid with that of topical fluocinolone acetonide in the treatment of OLP after 2 and 4 weeks of treatment, found that 0.1% fluocinolone acetonide reduced the severity of atrophic and erosive OLP better than 0.05% retinoic acid.^7^ The difference of that study with the present study was the use of zinc mouthwash instead of retinoic acid, but in both studies fluocinolone ointment decreased the severity of OLP lesions. However, further studies are necessary to exactly evaluate the reasons for the difference.

In a double-blind randomized controlled study on subacute and chronic eczema,^4^ lichen planus and limited psoriasis, 2.5% topical zinc sulfate in combination with 0.05% clobetasol propionate cream (Zincoderm Cream, Apex Laboratories) was found superior to topical steroid use alone, due to the anti-inflammatory properties of zinc sulfate by preventing the release of keratinocyte, an associated marker of inflammation. It also induces re-epithelialization of partial thickness wounds and second-degree burns.^4^ In the latter study, like ours, corticosteroid with zinc had a greater effect than corticosteroid alone.

The results of the present study are consistent with the findings of a study, which reported topical application of 0.5% zinc sulfate leads to improvement of the healing process. In comparison to controls, the granulation tissue is constructed more systematically, the formation of collagen is more advanced, and its amount is increased.^8^

An investigation of the effect of topically applied zinc on leg ulcer healing and examination of its effect on mechanisms in wound healing using standardized animal models, demonstrated that topical zinc oxide can promote cleansing and re-epithelialization and can reduce inflammation and bacterial growth.^9^ It has also been indicated that topical zinc oxide can increase endogenous gene expression of insulin-like growth factor-1 in granulation tissue. The increased gene expression of IGF-1 may be one mechanism by which topical zinc oxide enhances wound healing.^10^

## Conclusion

Within the limitations of the present study it can be concluded that use of zinc mouthwash with fluocinolone ointment is more effective than ointment alone in decreasing the lesion surface area.
